# 以乳酸酸中毒为突出表现的儿童急性淋巴细胞白血病一例

**DOI:** 10.3760/cma.j.issn.0253-2727.2021.12.017

**Published:** 2021-12

**Authors:** 雅琴 王, 东艳 崔, 必鑫 习, 文 余, 爱国 刘, 群 胡

**Affiliations:** 华中科技大学同济医学院附属同济医院儿童血液病科，武汉 430030 Department of Pediatrics, Tongji Hospital, Tongji Medical College, Huazhong University of Science and Technology, Wuhan 430030, China

患儿，女，6岁，因“发作性双下肢乏力1个月”于2017年9月18日入院。外院胸部CT正常。入院前多家医院诊断代谢性酸中毒、低钾血症和贫血，给予补碱、补钾和输血治疗后，酸中毒无好转，逐渐出现深大呼吸，伴腰痛，后收至我院儿童遗传代谢内分泌科进一步诊治。入院后查体：面色及口唇稍苍白，营养中等，呼吸深大，可见吸气性三凹征；心音稍钝，双肺呼吸音粗糙，未及明显啰音；腹软，稍胀，肠鸣音正常，肝肋缘下2～3 cm，质地韧，无压痛，脾肋缘下未及，腰骶椎处脊柱有压痛，四肢肌力正常。血常规：WBC 4.47×10^9^/L，中性粒细胞1.82 ×10^9^/L，HGB 61 g/L，PLT 129×10^9^/L。电解质示钾2.85 mmol/L，钠131.4 mmol/L，氯92 mmol/L，钙2.27 mmol/L，磷0.69 mmol/L，镁0.9 mmol/L。血生化示碳酸氢根5.4 mmol/L（正常参考值22～29 mmol/L），乳酸>15.5 mmol/L（正常参考值0.5～2.2 mmol/L），丙酮酸447 µmol/L（正常参考值20～100 µmol/L），余肝肾功能正常。血气分析pH 7.09，标准碳酸氢根5.8 mmol/L，全血碱剩余−24.5 mmol/L。阴离子间隙（AG）增高。彩超提示肝肋缘下2 cm；双肾体积稍大（左肾102 mm×45 mm，右肾109 mm×46 mm）。入院后给予纠正酸中毒、补钾及输血支持治疗，患儿酸中毒持续数天无好转。行骨髓穿刺检查，骨髓细胞学提示原始和幼稚淋巴细胞占52.8％，考虑急性淋巴细胞白血病（ALL）。流式细胞术免疫分型提示普通型B-ALL，融合基因示 MLL-AF4阳性，FISH示MLL（11q23）基因断裂重组阳性，染色体示t（4;11）。明确诊断为ALL（B系，中危）。后转至儿童血液病科，立即按照CCCG-ALL-2015方案开始规范化疗。口服地塞米松后，乳酸水平开始下降，在窗口期结束时，乳酸和血pH值接近正常，在化疗第7天，乳酸值恢复正常。患儿诱导缓解治疗后获得完全缓解，并且流式细胞术微小残留病（MRD）为阴性。在治疗第85周，患儿骨髓检查提示ALL复发。复发后RNA测序显示TP53突变，融合基因检测显示MLL-AF4阳性，提示预后不佳。随后该患儿接受了CD19嵌合抗原受体T细胞治疗（外院）和单倍体相合异基因造血干细胞移植，移植后获得完全缓解。2020年5月，患儿二次复发，再次接受了CD22嵌合抗原受体T细胞治疗（外院）并取得缓解，在等待二次移植过程中全面复发，最终治疗无效死亡。

讨论：儿童ALL可以顽固性乳酸酸中毒（LA）为突出表现，故非血液肿瘤科医师接诊LA患儿时，需将肿瘤性疾病作为鉴别诊断。对于急性白血病合并LA的患儿，化疗是改善LA的有效措施。既往文献报道，LA在肿瘤性疾病中提示预后较差。该患儿在治疗的早期阶段对化疗反应良好，直到治疗第85周患儿MRD持续阴性。但患儿初诊时MLL-AF4融合基因阳性，复发后RNA测序检测到TP53突变，此二者均提示预后不佳。故对于以LA症状为突出表现的儿童ALL，规范化疗也可以取得早期缓解，但其远期预后仍取决于疾病本身分子遗传学特点。

